# Experiences in Engaging the Public on Biotechnology Advances and Regulation

**DOI:** 10.3389/fbioe.2016.00003

**Published:** 2016-02-02

**Authors:** M. Megan Quinlan, Joe Smith, Raymond Layton, Paul Keese, Ma. Lorelie U. Agbagala, Merle B. Palacpac, Louise Ball

**Affiliations:** ^1^Centre for Environmental Policy, Imperial College London, London, UK; ^2^Advisor in Regulation, Science and Government (Formerly affiliated with the Office of the Gene Technology Regulator), Canberra, ACT, Australia; ^3^Industry Affairs and Regulatory, DuPont Pioneer, Johnston, IA, USA; ^4^Office of the Gene Technology Regulator, Canberra, ACT, Australia; ^5^Bureau of Plant Industry, Post Entry Quarantine Station, Los Baños, Philippines; ^6^Department for Environment, Food and Rural Affairs, London, UK

**Keywords:** GM, GM crops, GM animals, environmental risk assessment, communication, public engagement, consultation, regulation

## Abstract

Public input is often sought as part of the biosafety decision-making process. Information and communication about the advances in biotechnology are part of the first step to engagement. This step often relies on the developers and introducers of the particular innovation, for example, an industry-funded website has hosted various authorities to respond to questions from the public. Alternative approaches to providing information have evolved, as demonstrated in sub-Saharan Africa where non-governmental organizations and associations play this role in some countries and subregions. Often times, those in the public who choose to participate in engagement opportunities have opinions about the overall biosafety decision process. Case-by-case decisions are made within defined regulatory frameworks, however, and in general, regulatory consultation does not provide the opportunity for input to the overall decision-making process. The various objectives on both sides of engagement can make the experience challenging; there are no clear metrics for success. The situation is challenging because public input occurs within the context of the local legislative framework, regulatory requirements, and the peculiarities of the fairly recent biosafety frameworks, as well as of public opinion and individual values. Public engagement may be conducted voluntarily, or may be driven by legislation. What can be taken into account by the decision makers, and therefore what will be gathered and the timing of consultation, also may be legally defined. Several practical experiences suggest practices for effective engagement within the confines of regulatory mandates: (1) utilizing a range of resources to facilitate public education and opportunities for understanding complex technologies; (2) defining in advance the goal of seeking input; (3) identifying and communicating with the critical public groups from which input is needed; (4) using a clearly defined approach to gathering and assessing what will be used in making the biosafety decision; and (5) communicating using clear and simple language. These practices create a foundation for systematic methods to gather, acknowledge, respond to, and even incorporate public input. Applying such best practices will increase transparency and optimize the value of input from the public.

## Introduction

Public involvement is a critical part of the development, evaluation, and acceptance of any new technology. It has been considered to be especially important as part of the biosafety decision-making process and is included in the Cartagena Protocol on Biosafety (referred to below as the Protocol) to the Convention on Biological Diversity (CBD) (UNEP, 2000), specifically in Article 23. However, the inclusion of public participation in the Protocol is linked to the sovereignty of countries to determine how best to do that. Without details on the best approach,^1^ the Protocol leaves much to the terms and conditions of national biosafety frameworks and national decision makers (Mackenzie et al., 2003; Toczeck Skarlatakis and Kinderlerer, 2013).

A critical point is that most state-led engagement approaches the introduction of a novel technology in terms of public good and the mandate for maintaining biosafety. In the governmental sector, gaining broad support for the biosafety framework may occur as a political process, rather than as part of regulatory decision making (Wohlers, 2010). In an early review of the US biosafety framework for transgenic plants, one conclusion was that using risk analysis to make scientifically informed decisions regarding safety *as well as to legitimize the decision process* to the public creates a tension and can itself skew the process [emphasis added] (National Research Council, 2002). Using public consultation as a means to reduce risks posed to a project or outcome, including risk related to lack of public acceptance, can also backfire (State of Victoria, 2005). More encouragingly, engaging the public may provide a means of addressing long-held concerns, which had not even been articulated until the consultation, but which may be meaningfully addressed (Marris, 2001). These conflicting objectives and potential outcomes highlight what the authors observe to be an ever increasing tension between decision making based entirely on scientific evidence and decision making that is defined by societal values (Frewer et al., 2004; Ching, 2007). The integration of science and societal values is necessary for broader policy, but who are the actors and when is the moment to introduce considerations outside of scientific evidence?

This article aims to support those tasked with decision making by giving a general, pragmatic overview of practices for capturing and addressing public input regarding the decision process or to individual cases in the context of biotechnology. (The authors do not attempt to analyze larger questions on theory of public engagement, the case-by-case decision-making approach generally used, or national sovereignty in the details of public consultation.) It is widely agreed that there is no single best approach to public involvement in decision making, particularly for complex or controversial issues. The developers and/or introducers of a new technology, the overall government, and the regulators of these introductions may seek public input for various reasons. As laid out in Box 1, it is imperative to understand and define the purpose of any exchange on biotechnology, or other complex issues, before selecting an approach (Nuffield Council on Bioethics, 2012; Navarro et al., 2013; Biosafety Clearing-House, 2014a). Furthermore, the appropriate implementer of the public involvement process, and its timing and scale, are determined by the objective of the engagement and impacts on the outcome.

Box 1 **Fundamentals of public involvement.**

Effective engagement involves all important stakeholders including product developers, product users, and regulators – basically all persons or entities that could be directly affected by the new technology. In order to be effective, this engagement must be timely, transparent, and trustworthy. However, gathering public input for a case-by-case decision process may be driven by legislation that defines and sets the criteria for the extent and type of information that will be gathered, or the consultation may be conducted voluntarily.

The diagram below identifies this continuum of potential public involvement from (left) being a partner in an ongoing process by the applicant to gain and maintain a social license or to discover alternatives matching community preference through two-way communication and mutual learning; through the (center) opportunity for clarifying societal values through consultation on the decision framework, for example, and learning public opinion, with some educational efforts; through (right) a more traditional governmental call for comments within a set framework, to achieve compliance with consultation requirements at a set point in time. This continuum is from the public having significant influence on a decision to a more unidirectional communication of information and decisions. (The International Association for Public Participation (IAP2) schematic,^1^ the public participation spectrum used by the US Environmental Protection Agency (EPA),^2^ the State of Victoria (2005), and others, shows the continuum as moving, in a similar direction, from the steps to empower, collaborate, involve, consult, or inform.) The proposed response to the question of who is responsible to achieve these may be debated, but practical experience indicates that regulators should be increasingly involved in those consultations indicated in legislation but decreasingly involved in other formats.

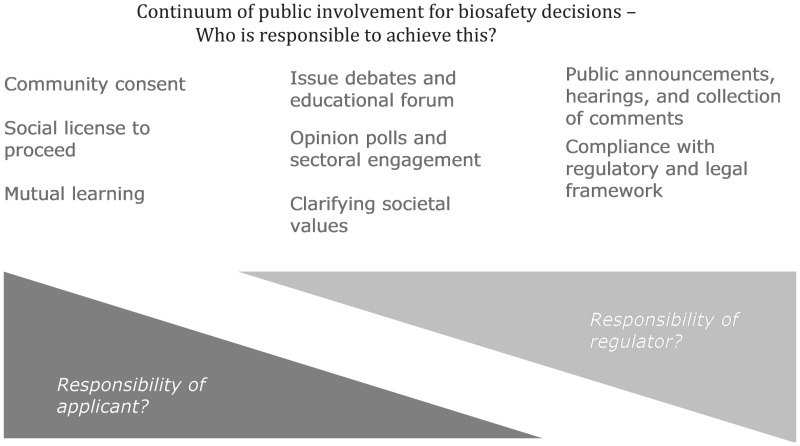

While these factors are well understood on paper, implementation of an effective public engagement plan is both complex and challenging. Complexities include communicating with multiple groups each of which enters the process at a different level of knowledge and understanding of the technology, biases, and sometimes a different set of goals for the interaction.

One of the greatest challenges is transparent and effective communication of both the potential benefits and potential risks of a product developed using a highly technical process to a public that may be skeptical of the benefits and risk averse. In fact, research suggests that case specific considerations can reveal nuances in public acceptance and concerns (Frewer et al., 2013). Other challenges include short timelines, inadequate funding, and limited expertise or experience in seeking, gathering, interpreting, and communicating public input. While the complexities and challenges of public engagement can be somewhat daunting, there are several examples of how they have been overcome to allow the biosafety decision-making process to move forward.

http://www.iap2.org/

http://www2.epa.gov/international-cooperation/public-participation-guide-introduction-guide

The discussion originated as presentations in a session on the same topic at the 13th International Symposium on Biosafety of Genetically Modified Organisms (Cape Town, South Africa, November 9–13, 2014), which was attended by a number of individuals responsible for regulatory decisions in their own country.

As background to this article, experts involved in public engagement were asked the questions in Table 1, which served as the basis for the presenters’ selection of cases. A broader survey of such individuals using these questions would provide further food for thought.

**Table 1:** **Questions to national policy makers, regulators, and developers or introducers of biotechnology on engaging the public**.

1. Why do you seek public input and what value have you derived from it?
2. What approaches have you found to be most effective in obtaining and addressing public input?
3. What have you found to be least effective?
4. What have been the most significant challenges you have experienced? And what guidance would you offer others on addressing these challenges?
5. What particular aspects of public engagement haven’t you worked out how to do yet?

Each of the experts presenting recognized the importance of systematic methods to gather, acknowledge, respond to, and, as appropriate, incorporate public input. Communication has been made more effective by translating scientific jargon to deliver a more understandable message. Structured approaches have been developed to more successfully gather and interpret public input and then communicate the results in a transparent fashion. Communications technology (use of the internet and social media) has been utilized to shift from a one-way communication of scientific data to a full dialog where the public can ask questions that are then answered by technical experts (Society of Biology, 2011).

The appropriate metric for gauging success of public involvement is not clear. Some argue that only participation that can lead to policy change is meaningful [e.g., Ching (2007)], yet when a framework is set, the value of participation is to play a role of broadening the perspectives included in the case in hand (Dietrich and Schibeci, 2003). Most agree that identifying, informing, and engaging with the public, and its many sectors, is the first step. The experiences and examples of practices for involving the public in decisions are more fully discussed in the three case studies presented in Section “Involving the Public in Decision Making.”

## Informing and Engaging the Public as a First Step

Communication with the public provides an opportunity to increase awareness and involvement of the broader community. A primary objective of this process is to help inform decisions with the experience and knowledge of a much wider group of “experts” than those included in a formal decision process (Dietrich and Schibeci, 2003; State of Victoria, 2005; Nuffield Council on Bioethics, 2012). There also is the hope that public understanding will increase public acceptance when a new technology has been evaluated and deemed safe by national biosafety decision makers, although this relies on an underlying trust of the system (Frewer et al., 2004; Sinemus and Egelhofer, 2007; Siegrist, 2008).

Developers and introducers of new technologies (the applicants in a regulatory system), clearly believing in the value of their technology or product, may employ public engagement as a way to gain support and acceptance of a technology either prior to or during regulatory review. In fact, the Convention on Access to Information, Public Participation in Decision-Making and Access to Justice in Environmental Matters, frequently referred to as the Aarhus Convention (UNECE, 1998), encourages information exchange between applicants and the public prior to official decision-making consultation. This practice varies considerably with the nature of the technology and the societal context into which it would be introduced.

### Engagement of Industry with the Public

Successful commercialization of genetically modified organisms (GMOs) requires not only regulatory approval but also public acceptance. Regulatory approval is obtained by following guidelines and submitting dossiers to government agencies. Establishing a consistent, coherent, and continuous two-way dialog between the developers of GMOs and the general public to help support public acceptance has been an elusive goal. In terms of communicating information regarding the safety of GMOs, industry, and other developers have typically used presentations at scientific meetings, publications in scientific journals, publicly available submissions made to regulatory agencies, and static online content. In most cases, communication efforts are made by individual developers, although groups, such as CropLife International and the Biotechnology Industry Organization, will sometimes provide information representing multiple developers. These efforts have met with varying levels of success depending on the topic and the audience but did not result in fully effective public engagement.

In 2013, GMO Answers^2^ was developed and then launched by a group of six companies (BASF, Bayer CropScience, Dow AgroScience, DuPont, Monsanto, and Syngenta) involved in the development of genetically modified (GM) crops as part of the Council for Biotechnology Information. In addition to the founding companies, there are 11 “supporting partners” – mostly crop associations that support the concepts behind the project. The goal of this new website and effort was to increase the level of conversation among interested parties. The website provides basic information about GM crops, links to studies and articles, and easy-to-read explanations and responses to frequently asked questions or topics. Perhaps, the most important function of the website (and associated Facebook and Twitter links) is that it provides an easily accessible location where questions about GM crops can be asked and answers are then provided by one or more experts. The experts include academics, trade organizations representatives, farmers, nutritionists, and developer company scientists. The source(s) of each answer is identified on the website. Questions that have been asked include not only those related to human and environmental safety, but also those regarding product development, economics, trade, and more.

As of March 2015, about 800 questions had been asked and answered on the website. In general, about 40% of responses are from industry experts, 30% from academic or other non-industry experts, and about one-quarter from the moderator (often because the question had already been asked and the query can simply be responded to by a link to the previous answer). While it is difficult to measure the direct success of this effort, the site has hosted 740,000 visitors since launching in 2013. In terms of broader and continued communication, the primary drivers for website traffic are links from Facebook and Twitter and approximately 30% of site users are returning visitors. Public engagement is expected to become broader as GMO Answers expands to other languages and communication pathways – for example, a Japanese language version of the GMO Answers website recently became available, other language sites are currently under development, and GMO Answers maintains both an active Twitter feed and Facebook page.

Where in the past, the primary engagement was between biotechnology developers and regulatory agencies, GMO Answers has begun to broaden the conversation so that it includes the general public. The act of responding to public queries, in addition to questions from regulators, increases both the breadth and depth of the interaction in a way that should increase public understanding, if not acceptance, of the technology. This is an example of improved involvement of the public, although education and access to information do not necessarily imply increased understanding and acceptance.

### Engagement by Non-Regulatory Biotechnology Organizations in Sub-Saharan Africa

Sub-Saharan Africa is in the early stages of developing regulatory frameworks for approving the commercial release of GM crops, including regulatory capacity to capture and address public input. One striking observation about sub-Saharan Africa from an external observer is the number of countries with public or quasi-public organizations or non-governmental organizations (NGOs), which were set up to provide information, education, or opportunities for engagement on biotechnology. The existence of these organizations, which are external to the regulatory framework, appears to be a result of varying factors: the emergence of the technology in relation to political contexts; the desire to consider potential benefits as well as risks to the countries involved from a perspective of scientific and human capacity, and socioeconomic development (McLean et al., 2012); and the lack of resources within regulatory bodies to manage any additional requirements of engagement.

There are several organizations that address issues of communication with the public as part of a broader remit to enhance biosafety capacity throughout the region. These organizations include the African Biosafety Network of Expertise under the New Partnership for Africa’s Development (NEPAD), the Open Forum on Agricultural Biotechnology in Africa, the Program for Biosafety Systems under the International Food Policy Research Institute, and the International Centre for Genetic Engineering and Biotechnology.

Regulatory biosafety legislation in many countries within sub-Saharan Africa has been shaped by the Cartagena Protocol and the African Model Law on Biosafety,^3^ which support the use of risk assessment for decision making but, in the case of the latter, from a particularly precautionary perspective. Nevertheless, the Protocol also acknowledges the tension between the need to protect human health and the environment from the possible adverse effects of the products of modern biotechnology and the potential of the technology to promote human well-being, particularly in meeting critical needs for food, agriculture, and health care. Ongoing African biotechnology research and the political support emerging for this has been summarized in recent publications (Karembu et al., 2009; Chambers et al., 2014), including the popular press. To generalize, historically the debate on policy has been largely influenced by both “pro” and “con” interests external to the region, as the general public relies on media to form opinions on complicated scientific questions. The existence of government and independent groups supporting education, communication, and stakeholder involvement is salutary to the degree to which the objectives and agendas of the organizations are made clear.

The International Service for the Acquisition of Agri-biotech Application has provided information on commercialization of GM crops since their first planting in 1996.^4^ Another early organization, set up close to the beginning of biotechnology commercialization, was AfricaBio in South Africa, a non-profit stakeholders’ association which plays the role of advocate of the potential benefits of technology, as well as promoting appropriate biosafety and regulation. A case study of AfricaBio (UNECE, 2013) acknowledged its good practices in involving local farmers as the facilitators, involvement of the entire rural community, and access to the yield results of the GM crop, in this case, maize. It was criticized for presenting information from one perspective. Although a self-stated advocate, it can provide scientifically based materials in an accessible manner for better understanding of biotechnology.

The Africa Harvest Biotechnology Foundation (AHBFI), established in Kenya in 2002, has acted in an advocacy role with often underrepresented stakeholders. The primary focus has been on delivering solutions to hunger and agricultural challenges by partnering, but more recently, a 10-year strategy has restated the need for deeper engagement beyond delivery of a transparent and scientifically supported message (AHBFI, 2012).

Other organizations are more like industry associations, such as the African Biotechnology Stakeholders Forum^5^ in Kenya. More quasi-public entities with ongoing government support, as well as other funders, include the Uganda Biotechnology and Biosafety Consortium (UBBC). The National Biotechnology Development Agency was established by the Nigerian government with the mandate to implement a policy specifically for “promoting, coordinating, and setting research and development priority in biotechnology for Nigeria”^6^; its mandate includes influencing future regulatory and legislative language.

This range of organizations forms a critical part of public engagement in their countries and regions, and, in some cases, global networks. The two decades since GM crop commercialization began also have seen significant changes in the role of the public in policy formation and regulatory considerations. As one publication (Navarro et al., 2013) puts it, science communication is not enough. These organizations in sub-Saharan Africa appear to be developing more locally appropriate methods for involving the public and encouraging input from the most critical groups affected by the decisions.

## Involving the Public in Decision Making

### Guidance on Environmental Risk Assessment for GM Animals in Europe

Presently, European legislation on field release of GMOs is covered in Directive 2001/18/EC of the European Parliament and of the Council of March 12, 2001 on the deliberate release into the environment of GMOs, and related regulations.^7^ The framework relies on the European Food Safety Authority (EFSA) to make scientifically based recommendations regarding applications (European Commission, 2015). This framework gives explicit directions only on assessing the risk from GM plants for import or cultivation for food or feed, but not yet on GM animals to be raised in the European Union territory.

In 2007, the European Commission requested that EFSA initiate a process to address environmental safety of GM animals, initially to complement guidance on risk assessment of GM animals in regard to food and feed (EFSA, 2012). The role of guidance documents has been to assist applicants in understanding requirements for applications, in line with the existing European legislative framework (EFSA, 2013a). Documents have been developed, or revised, as legislation has changed or experiences with applications have provided new insights. The development of guidance on environmental risk assessment involved review of existing materials, definition of the scope (including proposed categories and likely cases of GM animals ready for the European market in the near term), and identification of experts. Some materials were compiled by contracted consultants (Fera, 2010; Umweltbundesamt, 2010; University of Hull, 2010), and a draft Guidance outline of content was prepared by the EFSA GMO Unit.

New material was then developed by three working groups (WGs), convened for the purpose, over the course of around 2 years. The WGs focused on their respective areas of expertise in application to GM fish, GM mammals and birds, and GM insects, and jointly prepared material on the overarching and cross-cutting sections of the guidance. An important part of the process was a public consultation to allow stakeholder input, which was then considered by the same WGs, the relevant EFSA staff (the GMO Unit) and the EFSA GMO Panel. The EFSA GMO Panel endorsed the draft scientific opinion, which went to the public consultation process (EFSA, 2013b).

Consideration was to be given to all “scientifically relevant” comments, but not to risk management, risk/benefit analysis, and ethical and socioeconomic aspects raised in comments, which are outside the EFSA purview. The draft opinion was posted on the EFSA website,^8^ now available under closed consultations, and a comment period was open from June 21 to August 31, 2012. Efforts were made to publicize the process and encourage comment. A template and instructions for submitting comments supported the process. Specific criteria were given to define what would, and would not, be considered from public input: only submissions relevant to the document, within the deadline, on the template provided; no complaints against institutions, individual persons, or offensive material; and only comments related to policy within the scope of EFSA’s activity.

Twenty-five entities – including national governmental organizations and NGOs, research institutes and universities, industry groups, and individuals – submitted 720 comments in response to this opportunity. All comments were published (EFSA, 2013b), including those which did not meet the stated criteria for receiving consideration. Only 10% appeared to be repetitions of a phrase likely provided by a single source to those submitting: “EFSA is not competent to assess environmental risks as it has no remit or expertise in this area” or similar wording. The identity of the stakeholder was published, except when an individual person, in which case the comments were included but the individual was anonymous. The vast majority of the 720 comments arose from within the European Union Member States, with half identified as from Germany (195 of which were from the Max-Planck-Institut für Evolutionsbiologie – the Max Planck Institute for Evolutionary Biology) and one-third from the UK (143 of which were from GeneWatch UK). Comments were also received from individual(s) listed as from the USA, two US NGOs, and an agency in the Government of Canada.

A significant portion of the entire time involved in developing guidance went into considering and responding to the public input. The EFSA GMO Unit (staff) organized the comments for the WGs and therefore sorted the relevance and topic prior to consideration by the WG experts. However, the WGs invested many hours reviewing and rewriting in response to the comments. Significant improvements to the guidance included clarification of the scope to include both intentional and accidental release to the environment within the European Union (but not confined use in general), greater consistency of terminology and concepts across the various chapters, enhanced guidance on the environmental risk assessment step of problem formulation and clarity of objectives of each step, and closer alignment of the guidance to the relevant European legislation (Directive 2001/18/EC) (European Commission, 2001), as well as better explanation of sections considered unclear (EFSA, 2013b).

In summary, this process of developing guidance for assessing potential risks from the introduction of GM animals to the environment (EFSA, 2013a) included a significant effort to engage with the affected national governments and public, but also allowed comment from outside the European Union. The entire process took just over 6 years. The improvement in the document was acknowledged by EFSA, but opponents to any GMOs or those who question EFSA’s role in preparing guidance will remain frustrated since the forum was not meant to address the overall biosafety framework which assigns EFSA its role.

This stands in contrast to the 2003 “GM Nation?” consultation covering the UK in several ways: the UK consultation was to allow public comment on the overall issue of commercializing biotechnology; the eventual effect of the public input was not clearly defined in advance; and participation was largely self-selected (as with the EFSA consultation) but was supplemented by focus groups of more representative populations (although these were later found to be methodologically imperfect) (Pidgeon et al., 2005; UNECE, 2013).

The ongoing collection of input on the policy and framework (although external to the regulatory body) described below for Australia, and the process led in New Zealand by the Royal Commission on Genetic Modification (2001) have attracted wide participation over time. The latter collected over 10,000 written comments and numerous opinions at public workshops to conclude that the country should proceed cautiously with the introduction of GMOs due to public concern. Such a process is designed to elicit public opinion on broader questions than the specifics of risk assessment or particular cases. The purpose of the EFSA guidance was to clarify what information would be required from applicants in order to evaluate a release of a GM animal into the environment in the territory of the European Union and how that information would be considered. Comments on the overall policy, legislative framework, and competency of each component of the framework were irrelevant to the consultation yet dominated the submitted comments.

### GM Crops in the Philippines

The Philippines has been heralded as the first Asian country with a biosafety regulatory framework, which was put to public consultation at the time (NAST, 2009), and commercial approval of a major GM crop (Panopio and Navarro, 2011). The Philippine government’s policy statement^9^ makes clear the official vision for biotechnology: “We shall promote the safe and responsible uses of modern biotechnology and its products as one of the means to achieve food security, equal access to health services, a sustainable and safe environment and industry development.” While the regulatory system has been challenged, it has generally worked due to the commitment to scientific criteria and transparency in decision making (Halos and Soriano, 2014). For example, a website was established early on to facilitate public access to documents related to applications (NAST, 2009).

However, some local government units (LGUs) took a negative stance against GMOs, even going so far as to declare themselves as GMO-free zones (Cabanilla, 2007). The legal authority for LGUs to have autonomy from national government in some aspects was given by the Local Government Code (Republic Act 7160 of 1991), which coincidentally coincided with the early field trials of GM crops. This division of authorities, along with involvement of international NGOs, has complicated the public engagement process and led to varying views on its legitimacy. Increased capacity building for local government is considered one way to improve understanding at that level in the future (NAST, 2009).

#### GM Corn

The first confined field trials in the Philippines were for transgenic corn, conducted under different permits by two international corporations. As is often the case, the evolution of the regulatory system and development of the steps toward commercial release were spurred on by these early applications (NAST, 2009). The GM corn cases were also cited as a turning point for the National Committee on Biosafety in the Philippines (NCBP) in their understanding of the range of stakeholders interested in their decisions. While initial engagement was with the applicants – developers and introducers of the advances in biotechnology – it was the opponents or the public at large who required in depth education and communication opportunities. This was a shift in approach for the NCBP (NAST, 2009).

Experiences with field trials and regulatory approval of GM corn are described by Panopio and Navarro (2011) as a “drama.” Cabanilla (2007) refers to the protests and destruction of crops during the period when GM corn was in confined field studies as being conducted by “militant groups,” listing those as Kilusan ng Magbubukid sa Pilipinas (KMP, literally translated as Peasant Movement of the Philippines); MASIPAG (acronym for Magsasaka at Sayantipiko Para sa Ikauunlad ng Agham Pangagrikultural), South East Asia Regional Initiatives for Community Empowerment (SEARICE), Greenpeace, and the Philippine Greens. However, these more radical positions did not take away the interest in the production sector. Presently, six transgenic events of corn have been commercially released in the country. In <10 years after the first commercial production of GM corn was approved, the Philippines was reported to be a self-sufficient producer of corn and one of the larger producers of GM crops globally (Fernandez, 2015).

#### Bt Eggplant

One of the more publicized cases in apparent public reaction to regulatory approval of a GM crop is *Bacillus thuringiensis* (Bt) eggplant or, as locally known, Bt talong. In the Philippine biotechnology regulatory system, public information and participation applies to all stages of the biosafety decision-making process starting from the time the application is received. Early on in the process, Institutional Biosafety Committees are required to be established in institutions involved in genetic engineering research. A committee must be composed of at least five members – at least three scientists and two community representatives who will represent the interest of the surrounding community with respect to health and protection of the environment. Institutions applying for field testing of a GMO are required to notify and invite the public to give comments on the proposed activity. A Public Information Sheet (PIS) about it must be posted for at least 3 weeks in three conspicuous places in the barangay (village) where the study is to be conducted. For application for propagation, the information sheet must also be published in two newspapers of general circulation.

The initial public engagement for this case was aimed at informing the public of the upcoming regulatory decision and the potential benefits of the approval for Bt eggplant multiple field trials. The contrast between using this innovation and the dangers of continual pesticide use was documented clearly [e.g., Panopio and Mercado (2010)]. Farmers facing the pest problems were reported to be supportive and eager for the crop to be commercialized (Subbaraman, 2011).

Campaigns against the trials were conducted by Greenpeace Southeast Asia, the *Magsasaka at Siyentipiko para sa Pag-unlad ng Agrikultura* (Farmer-Scientist Partnership for Development – its acronym, MASIPAG, means active or energetic in Tagalog) and *Sibol ng Agham at Teknolohiya* (Wellspring of Science and Technology, SIBAT). The collection of public opinion has been documented to be heavily influenced by external antibiotechnology interest groups. Their efforts, which culminated in the Philippines Court of Appeals stopping the trials in May 2013, relied largely on greater resources and organization than the proponents of the technology possessed (Laursen, 2013).

Analysis suggests that the focus on the technology for achieving a beneficial outcome and the scientific advance it represented were ineffective for achieving public understanding of the risk/benefit involved (Escano, 2013). Messages were considered to be too technical. The message of further documentation of the benefits to farmers [e.g., Gerpacio and Aquino (2014)] was possibly lost, as the pesticide regime is a familiar risk and the fear of the unfamiliar was a key point in opposing media. As the legislative framework did not limit public input to those affected by the technology and there is no mechanism for understanding the source of changes in opinion, there is nothing to prevent this situation. Rather than relying on scientific debate, the input from Greenpeace, for example, has been categorized as playing on emotions (Ropeik, 2013).

In this case, the legislative framework almost appears to have worked against the public input process. National legislation requires informing the public of the conduct of the field trials by posting a PIS in areas where the trials will be done. Several trials were attacked, damaging the research study. The first delay in trials was based on charges that the public consultation was not accomplished according to law, although the variation from requirements was very minor (Ilano, 2011). Competing jurisdictions between national, regional, and municipal confused the process to some extent.

Since the Court of Appeals’ judgment stopping trials, there have been public consultations and meetings with farmers to attempt to progress scientific understanding and document local acceptance.^10^ In 2014, the Biotechnology Coalition of the Philippines was allowed to introduce a petition to the High Court noting, “Greenpeace does not have assets or properties affected by the Bt talong field trials. Greenpeace has no actual, direct and immediate stake in the subject of the litigation.”^11^ The decision of the Supreme Court of the Philippines on the Motion for Reconsideration filed by the respondents to the case is still pending at the time of this article.

The Philippine regulatory agencies have increased the interactions with stakeholders and allowed concerns raised by various groups to be introduced into the consultation process. However, they have also remained clearly committed to a science-based risk assessment and decision-making process. The public good of the benefits to the farmers is considered much greater than the cost, in all senses, of the regulatory process (Halos and Soriano, 2014). The challenges of this country’s approach would not recommend it as a model of success, yet stakeholders have had a voice, even through the court system, at the time of formation of the biosafety framework and through to the case-by-case decision system today.

### The Australian Perspective

The Australian approach to communication with the public about the release of a GMO into the environment is determined by legislation and by administrative processes established to achieve consistent, sound regulatory decisions.

The Australian gene technology legislation requires informing the public on the regulatory decision-making process and seeking public input into that process. These aspects of openness and transparency, together with consultation, are intended to build trust in the regulatory system. Therefore, communication extends to a broad range of stakeholders, such as the license (or “licence” as indicated in Australian legislation) applicant/holder, the Gene Technology Technical Advisory Committee, the Minister for the Environment, other federal regulatory bodies, and State and Territory governments, as well as the public. Reviews and consultations on the legislation and policy governing the Australian regulatory scheme are conducted by a policy section of government under the oversight of a Federal/State Ministerial Forum. The regulatory body, the Office of the Gene Technology Regulator (OGTR), is responsible for administering the regulatory scheme, as well as advising the policy makers on its effectiveness, possible improvements, and other technical questions.

Public input is achieved through engagement at public meetings and access to the regulatory agency via a toll-free telephone number, email, and letter. Nevertheless, the primary means of communication is through submissions sought on a consultation version of the main document used in decision making, namely, the risk assessment and risk management plan. This document is prepared for each license application to approve a field trial, clinical trial, or commercial release of a GMO.

Development of efficient administrative processes to support decision making has led to applying similar approaches and language for seeking public input, irrespective of the type of organism, novel trait, or end use. However, public responses to different license applications may vary in unexpected ways. This is illustrated by contrasting two GMOs that were approved for environmental release. One case concerns the commercial release of GM cotton throughout Australia, while the second case relates to a clinical trial of a GM vaccine for cholera.

#### GM Cotton – Public Submissions

In 2014, a consultation risk assessment and risk management plan for a commercial release of GM cotton with insect resistance and herbicide tolerance was prepared and public input sought over an 8-week period.^12^ Only four submissions were received. Three submissions opposed approval of the release, while one supported its approval. Although this number of submissions is low, it is consistent with the relatively low concern expressed about all GM cotton license applications. In addition, the types of comments in the three submissions that did not support approval raised concerns similar to those raised for other GM cotton releases, including lack of confidence in the applicant’s data and the reliance of decision making on that data; request for additional information on potential adverse effects; lack of information on long-term and cumulative effects; possible effects on bees and other beneficial pollinators; purported insufficient evaluation of health and environmental safety issues; and concerns about increased herbicide use.

#### GM Cholera Vaccine – Public Submissions

A consultation risk assessment and risk management plan for a clinical trial of an oral GM vaccine for cholera limited to a maximum of 1000 volunteers was also released for public input in 2014.^13^ In contrast to the GM cotton case, 68 submissions (all opposing the trial) were received, as well as many direct calls to the regulatory agency and many comments on social media. This level of response was unexpected as GMOs used as human therapeutics (e.g., GM vaccines) usually raise less concern than GM crops.

Some of the concerns included opposition to vaccination in general; lack of relevance due to limited occurrence of cholera in Australia; potential for horizontal gene transfer; purported insufficiency of data; the possibility of inadvertent exposure of people to the GM vaccine through disposal into sewage and dump sites (i.e., through nappies); issues with the design of the trial (e.g., ethics of using children, long-term effects, compensation issues, meaning of results, or need for independent oversight of participants’ safety); and disputing the need to keep certain commercial information confidential.

Some of the marked interest in this particular license application can be attributed to a message in social media, which erroneously claimed that there would be aerial spraying of the vaccine. This message was amplified primarily among anti-vaccination groups, but did not appear in mainstream media. In addition, the use of technical/bureaucratic language may have obscured the message.

#### Public Input to Case-by-Case Decisions Informing the Decision-Making System

Consultation with the public on applications for the proposed environmental release of a GMO has been successful in providing the public with the opportunity to contribute directly in the decision-making process. As demonstrated by the two cases illustrated above, the number of responses can vary substantially according to the type of GMO and level of concern.

In addition, expression of the varied concerns, issues, and values of the public contributes to improvements in regulatory decision making. Some of the major lessons that have emerged from public input to decision making on GMO releases during 14 years of operation under the current Australian regulatory system include

- Only a small proportion of the Australian public has expressed concerns through submissions on individual license applications, but these mirror the level of concern reported in government surveys on public attitudes to biotechnology.
- Many concerns fall outside the scope of the legislation.
- Many concerns do not accord with the facts but reflect certain values and world views.
- The public is a conglomerate of distinct groups with different interests, concerns, and preferred channels of communication.
- Greater effort is required to communicate with the public in simple, clear language.
- Openness, transparency, and accessibility need to be demonstrated in meaningful and practical ways.

The Australian process varies from many others in its ongoing consideration of issues regarding the framework and not only the current case decisions. It may be worth noting that the OGTR is staffed by over 40 full-time-equivalent staff. The resources are greater than in many developing countries.

## Discussion

Public input to biosafety decisions can be a valuable resource for improving risk assessment, communication, and risk management. Yet trying to use public engagement as a means to legitimize the process can be counterproductive. For whichever objective, public input is sometimes a painful process. The need to capture and address public input is a responsibility under the Cartagena Protocol (Article 23) and most national biosafety frameworks. Specific guidance on how to achieve this is not provided, however. The responsible parties for each step along the continuum of involvement and the approach to consultation are dictated by the national biosafety framework, the available financial resources, capacity, and interest (UNECE, 2013).

Due to the complexity of many of the issues, public education and enabling initiatives can play an important role in supporting input to biosafety decisions (Rollin et al., 2011). Realistically, few regulatory agencies can serve as educator, communicator, and regulator. The applicant, who is usually the developer of the technology, is a necessary source of information in order to ensure accuracy about the technology or product and the intended study, release, or commercialization plans. A two-way dialog between industry and the public facilitates improvement in risk communication and may highlight areas for improved assessment or management. The landscape of biosafety information and education sources in sub-Saharan Africa has shown the need for parties outside the confines of regulatory agencies to provide ongoing education and representation of stakeholders, particularly when societies are eager for benefits that address basic health, food security, and economic development, but may be open to external influences.

The importance of defining in advance the goal of seeking input cannot be overemphasized. Equally, it is important to draw a distinction between public engagement for formation of policy and development of the decision-making process and public input to a specific case within that framework. Frequently, comments are submitted on the former when the consultation is addressing the latter. For those decisions which do relate to the policy or overall decision framework, such as the guidance on risk assessment of GM animals in Europe or on the decision process in Australia, enhanced decision-making frameworks may have long-term effects.

The cases discussed show how interest groups, often external to those stakeholders affected by a decision, can use the opportunity for public input as a battlefield to advance their values. This has certainly been the case for the Philippines where the biosafety framework is open to input from any sector, and NGOs have used legal means outside the framework to cause delays to approved trials for a GM crop which, if deployed, could contribute to a marked reduction in use of chemical pesticides. Herring (2008) in a perspective piece referred to the “archetypal deep disjuncture between the ideas and interests of global activists and the farmers they claim to represent.”

Those faced with decision making within a prescribed framework will benefit from criteria and guidance on who constitutes the public to target for consultation.

Reaching the public and gathering input has many challenges but can be achieved through various means. The government then must establish and implement a logical and transparent system for what it will require and how it will evaluate the scientific data that form the basis of a sound biosafety decision. Guidance to regulators on the scope of feedback to consider is crucial. Otherwise, the process can be dominated by value-laden viewpoints that cannot be affected by new information, education, or engagement. As Rollin et al. (2011) note, in some cases “information activates already existing attitudes, rather than changes them.”

Finally, conclusions and responses to consultation should be communicated back in an accessible manner. This serves to inform the public but also builds trust in the process, for those taking the time and trouble to provide input. The fact that this remains a weak link in most experiences indicates that further work on effective communication of this type would be beneficial. An interesting side note to this is the consideration of public involvement in ongoing monitoring of risks and benefits, which also requires effective feedback mechanisms operating over time (Ching, 2007).

In conclusion, several general practices have proven to be effective for involving the public in decisions on specific cases: (1) utilizing government and independent groups to facilitate public education, communication, and involvement about complex technologies beyond usual public understanding; (2) defining in advance the goal of seeking input; (3) identifying the critical public groups from which input is needed, and effectively communicating to these both the consultation goals and how input will be gathered and used; (4) using a logical, transparent approach to gathering and assessing scientific data that will be used in making the biosafety decision; and (5) communicating findings and response to input using clear and simple language. These practices create a foundation for systematic methods to gather, acknowledge, respond to, and even incorporate public input. Applying such best practices will increase transparency in terms of the scope and limits of the consultation and will optimize the value of any input from the public. This also reduces the sociopolitical pressures on regulatory decision makers who are working within an already established framework, thereby allowing decisions to be made based on the criteria under their remit.

It has been observed that a number of countries treat actions or scope suggested in the Cartagena Protocol, or in other guidance developed outside the process of the CBD Conference of Parties [e.g., UNECE (1998, 2003)], as legally binding requirements within their own national legislative framework. This can lead to onerous expectations. In fact, it can be counterproductive to attempt public participation without sufficient resources to conduct the process, capacity among both facilitators and the participants, ability to design a proper process which offers transparency, and a climate of integrity.^14^ Financial resources for implementing Article 23 are considered to be insufficient in many cases (Biosafety Clearing House, 2014b). In reality, it is often the resources available that determine the approach. This has been addressed to some degree through creative coalitions, public and private mechanisms for providing educational materials, and opportunities for public awareness raising and involvement. Supporting these broader processes also may alleviate the pressures on regulators who, the authors believe, should be limiting their deliberations to science within their regulatory mandate.

## Conflict of Interest Statement

Authors’ routine duties involve some of the suggested practices described, but no personal gain or influence in any author’s favor is connected with this publication or with the uptake of particular recommendations. The reviewer, Morven McLean, and handling editor, Andrew F. Roberts, declared their shared affiliation, and the handling editor states that the process, nevertheless, met the standards of a fair and objective review.

## Footnotes

^1^Recent work through the Cartagena Protocol process on public participation in biosafety decisions, including comments on best practices, is available at the Biosafety Clearing-House. This is summarized at http://bch.cbd.int/protocol/cpb_art23_info.shtml

^2^http://www.gmoanswers.com

^3^http://hrst.au.int/en/biosafety/modellaw

^4^http://isaaablog.blogspot.co.uk/

^5^http://absfafrica.org/

^6^http://www.nabda.gov.ng

^7^In order to rationalize the values expressed by individual Member States which are blocking field release for reasons other than human safety and the environment, with the implementation of the biosafety framework (European Commission, 2015), the Commission has now added Directive (EU) 2015/412 of the European Parliament and of the Council of 11 March 2015 amending Directive 2001/18/EC as regards the possibility for the Member States to restrict or prohibit the cultivation of GMOs in their territory (OJ L 68, 13.3.2015, p. 1).

^8^http://www.efsa.europa.eu/en/consultationsclosed/call/120621.htm

^9^A full copy of the policy statement appears at http://www.bic.searca.org/info_kits/policies/policy_statement.htm

^10^http://www.businessmirror.com.ph/farmers-plead-with-sc-junk-case-vs-bt-eggplant/

^11^http://www.interaksyon.com/article/95059/sc-allows-biotechnology-group-to-intervene-in-bt-eggplant-case---bcp

^12^http://www.ogtr.gov.au/internet/ogtr/publishing.nsf/Content/DIR124

^13^http://www.ogtr.gov.au/internet/ogtr/publishing.nsf/Content/DIR126

^14^http://www2.epa.gov/international-cooperation/public-participation-guide-introduction-guide
